# Enhancer-promoter interaction facilitated by transiently forming G-quadruplexes

**DOI:** 10.1038/srep09165

**Published:** 2015-03-16

**Authors:** Hedi Hegyi

**Affiliations:** 1 CEITEC—Central European Institute of Technology, Masaryk University, CZ-62500 Brno, Czech Republic

## Abstract

G-quadruplexes are guanine-rich nucleic acid sequences capable of forming a four-stranded structure through Hoogsteen hydrogen bonding. G-quadruplexes are highly concentrated near promoters and transcription start sites suggesting a role in gene regulation. They are less often found on the template than non-template strand where they either inhibit or enhance transcription, respectively. However, their potential role in enhancers and other distal regulatory elements has not been assessed yet. Here we show that DNAse hypersensitive (DHS) cis-regulatory elements are also enriched in Gs and their G-content correlate with that of their respective promoters. Besides local G4s, the distal cis regions may form G-quadruplexes together with the promoters, each contributing half a G4. This model is supported more for the non-template strand and we hypothesised that the G4 forming capability of the promoter and the enhancer non-template strand could facilitate their binding together and making the DHS regions accessible for the transcription factory.

G-quadruplex, a motif of four runs of three or more consecutive guanines, forming a stable tetraplex structure, is increasingly recognized as an important non-B DNA local structure in the regulation of gene expression^1^. G-quadruplexes are considered to be functionally important in all forms of cellular life, from bacteria^2^ to yeast^3^ to human^4^, both in DNA and RNA but their rapid evolutionary expansion was observed starting in metazoan organisms^5^. A growing body of evidence implicates them in various cellular functions such as transcription and translation regulation^6^, telomere maintenance and genome stability^7^. They are mostly found in promoters^8^, 5-UTR regions^6^ and to lesser extent also in gene bodies^7^.

While the regulatory role and formation of G4s in the promoter regions has been fairly well established, relatively little is known about their putative function in distal cis regulatory elements, such as enhancers and silencers^9^. The only G-quadruplex found so far in cis regulatory elements was identified in the 5' untranslated region of MECP2 mRNA, a gene associated with autism^10^.

Another unsolved question of gene regulation is how enhancers and promoters interact in transcription activation at a long distance, often spanning 1–2 Mbases. In recent years it has become clear that to do this, enhancers come into close proximity with target promoters with the looping away of intervening sequences^11^. In a few cases proteins that are involved in the establishment or maintenance of these loops have been revealed but how the proper gene target is selected remains a mystery^11^. Transcription factors or their complexes are thought to mediate enhancer-promoter loop formation but the proteins involved have been functionally identified in only a few cases^12^.

In this paper we investigated the presence of G4s, half-G-quadruplexes (i.e. two runs of guanines, both containing at least three consecutive G-s) and also transcription factor-binding sites in the nearly 600,000 regulatory cis elements in the human genome regulating the expression of all known genes, both protein- and RNA-coding, identified by Thurman et al.^13^ in the ENCODE series of papers. We found that G4s are about half as frequent in these DHS cis regions as in the DHS promoters, both taken from Ref. 13.

We also found that half G-quadruplexes (we dubbed G2s) were significantly enriched at the boundaries but depleted in the vicinity of these DHS sites.

After eliminating several possible causes for the enrichment of guanines and especially G2s at the boundaries of DHS sites in both promoters and cis elements we concluded that besides forming local G4s, they could also promote the interaction of the two regions by binding to each other and forming G-quadruplexes together.

## Results

### Half G-quadruplexes (G2s) in promoters and cis elements

**Figure 1:**
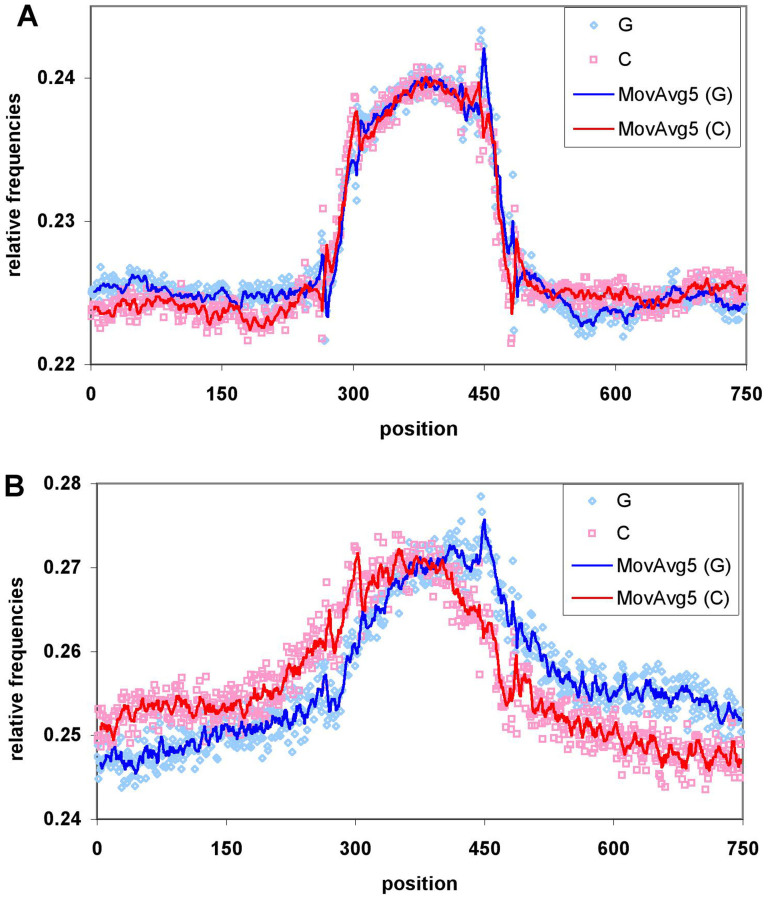
The relative frequency of guanines (Gs) and cytosines (Cs) in the DNase hypersensitive (DHS) sites in (A) 580,000 cis regulatory elements and (B) 63,000 promoters and their 300 nt-long flanking regions in the human genome identified by Stamatoyannopoulos et al.^13^ The actual DHS regions are located in the 300–450 nucleotide range. The dots indicate the actual numbers whereas the lines indicate moving averages of five values at each position, throughout the paper.

At first we counted the individual nucleotide frequencies in all the 63,000 promoter DHS sequences and also in the 580,000 correlated cis regions (enhancers and other regulatory elements) determined in Ref. 13. In Figure 1 their relative frequencies are shown for the actual 150 nt-long DHS sites and in their immediate vicinity of +-300 nucleotides (after extending them in both directions). Apparently, both the promoter and cis regions 5′ boundaries are significantly enriched in G-s and C-s, with a spike for G-s at the 3′ boundaries and for C-s at the 5′ boundary.

**Figure 2:**
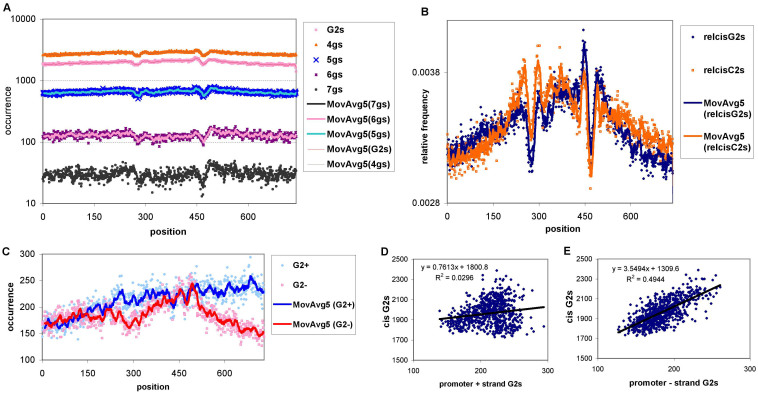
Distribution of G- and C-rich patterns in the cis elements and promoter regions. (A) G-tuplets of various length and G2-s (two runs of at least 3 consecutive G-s). (B) G2s and C2s in the cis elements and their 300 nucleotide vicinity. All tuplets and patterns were counted on the positive strand of the reference human genome throughout the paper. (C) The occurrence of the G2 patterns in the DHS promoter regions, separately for the (+) and (−) strand coded genes. Scatter-plot representation and correlation (R^2^) between the DHS cis elements (as shown for the 750 nt-long regions in (B) and (D) G2 occurrence on the (+) strand promoters; (E) G2 occurrence on the (−) strand promoters.

In the next step we counted patterns of consecutive guanines with increasingly longer runs of Gs and also patterns of two runs of at least 3 consecutive Gs with 1–7 nucleotides of any type between them (i.e. patterns of G2s). The results for the cis regions are shown in Figure 2a. Clearly, the boundaries are enriched and the vicinities depleted for all runs of Gs and also for **G2s**. In Figure 2b the distribution of **G2s** and **C2s** (two runs of minimum 3 consecutive Cs) are shown together for the cis regions. Apparently, the distribution of **C2s** is similar to that of the G2s and symmetric to the middle of the DHS regions, reflecting a potential role for G2s on the negative strand (every cis region in the study was defined on the positive strand).

The promoter regions were similarly analyzed (Figure 2c) but here we could distinguish between the promoters of positive and negative strand-coded genes. As the number of promoter regions in the Thurman data set is about an order of magnitude smaller than the number of the cis regions (63,000 vs. 580,000), the enrichment/depletion pattern for the former is apparently noisier than for the cis elements. However, there is also a significant enrichment of G2-s for the promoters at the 3′ end of the DHS regions, especially for the promoters of genes coded on the negative strand. Plotting the position-wise values of G2s between the cis regions and both the positive and negative strand promoters for the entire 750 nt-long regions revealed (Figure 2d, e) that while there is only a relatively small correlation between cis region values and the positive strand promoters (r = 0.248), there is an almost 3 times stronger correlation value of 0.709 between the cis region and the negative strand promoters' position-wise G2 values (Figure 2e).

### Local G-quadruplex forming potential

**Figure 3:**
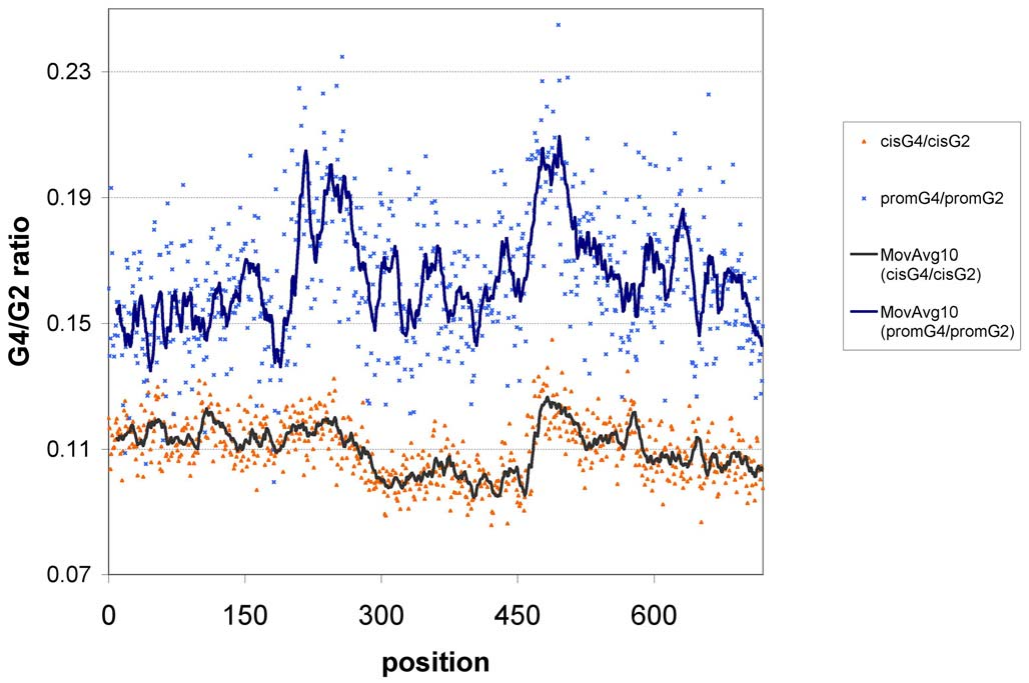
Position-wise ratios of G-quadruplex (G4) and G2 pattern occurrences for the promoters and cis elements.

To see if the sole purpose of the observed G2 patterns in the cis and promoter regions is their capacity to form local G-quadruplexes we calculated the position-wise occurrence of the latter for both the cis and promoter regions and divided them by the position-wise number of G2s separately for the cis regions and promoters (Figure 3). Using Student's t-test we found that both at the 5′ and the 3′ boundaries of the DHS regions the G4/G2 ratios are significantly higher than in the actual DHS regions (p-value < 1e-5), indicating a decreased propensity to form local G-quadruplexes in these regions, both for promoters and cis elements. Interestingly, plotting the G4 values against the G2 values for the 750 data points (Supplementary figure 1) revealed that for the promoter regions G4 values have a better correlation with the corresponding G2 values (Pearson correlation coefficients: 0.726 for promoters, 0.478 for cis-elements, p-value < 1e-6 for either correlation) than for the cis elements. Comparing Figure 3 with the plots of G2 enrichment for the cis elements and promoters (Figure 2b, c) also reveals that while the G2 enrichments are at the exact DHS boundaries (at positions 300 and 450), the G4 enrichment maxima are about 30–50 nucleotides shifted sidewise from the DHS boundaries both for the cis elements and the promoters. These observations might indicate a role for the cis G2 enrichment patterns beyond the local G-quadruplex forming capability at the DHS boundaries.

### TFBS in the DHS regions

**Figure 4:**
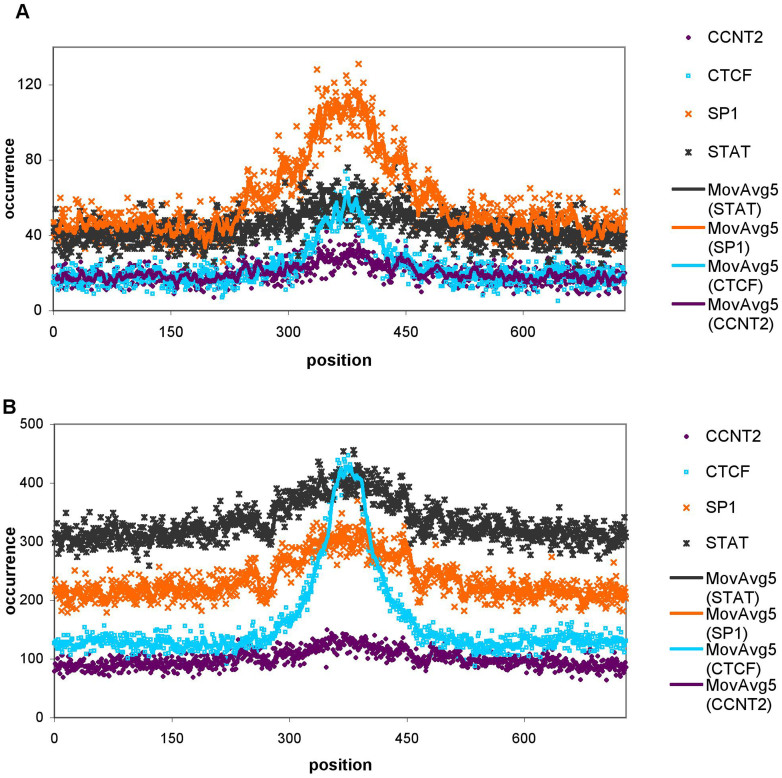
Transcription factor binding sites (TFBS) in the DHS promoters and cis elements. Position-wise occurrence of four of the most prevalent TFBSs mapped to the (A) promoters and (B) cis elements using the dataset from Ref. 14.

As DHS sites are known to bind transcription factors, we further queried the cis elements and promoters for transcription factor binding site (TFBS) motifs. We used the dataset of Kheradpour & Kellis^14^ who carefully mapped all known TFBSs to the entire human genome combining five different methods. In principle we mapped all TFBSs identified in Ref. 14 to the cis regulatory elements and promoters to see if the G-enrichment patterns in and near the DHS sites can be related to transcription factor binding. Figure 4 shows four of the most frequently occurring transcription factor binding sites in and near the DHS sites for both the cis elements and promoters. With the exception of CTCF all transcription factors have small local maxima at the boundaries of the DHS sites, and all four show a modest but significant correlation (Pearson correlation values between 0.37 and 0.56, Supplementary Table 1) between with the G2 patterns for both the cis elements and the promoters, however, they apparently do not account for the entire G2 enrichment patterns as their numbers are about an order of magnitude less than the numbers of G2s and they are also significantly more enriched in the DHS regions than the G2 patterns. The latter is especially pronounced for the SP1 patterns in the promoters shown in Figure 4a. Interestingly, while the 5′ boundaries (in a region of +- 50 nucleotides around the 5′ DHS region boundaries) show the strongest correlation for both the promoters and the cis elements between G2s and TFBSs, the 3′ boundaries show such correlation only for the cis elements but not for the promoters.

In Supplementary figure 2 the total number of annotated TFBSs^14^ are shown, together with the total number of predicted G2s. While they have similar patterns, and a significant overall Pearson correlation (0.628) over the entire extended region of 750 nucleotides, they also cannot explain all the G2 enrichment. Firstly, for the more critical region of the middle 250 nucleotides the correlation between the two drops to 0.346. In addition, we also calculated for each position the ratios of G2s/TFBSs and the reverse ratios of TFBS/G2s (shown in Supplementary figure 2d & e). While the G2/TFBS ratios show two marked local maxima at the exact boundaries (at around 300 and 450 in the figures) of the DHSs, for the opposite ratios of TFBS/G2s the exact boundaries show local minima while at about 20 nucleotides up- and downstream two marked maxima are shown. This provides further support that the G-rich regions and specifically the G2s have a role that is independent of transcription factor binding.

### Promoter-cis region correlation

**Figure 5:**
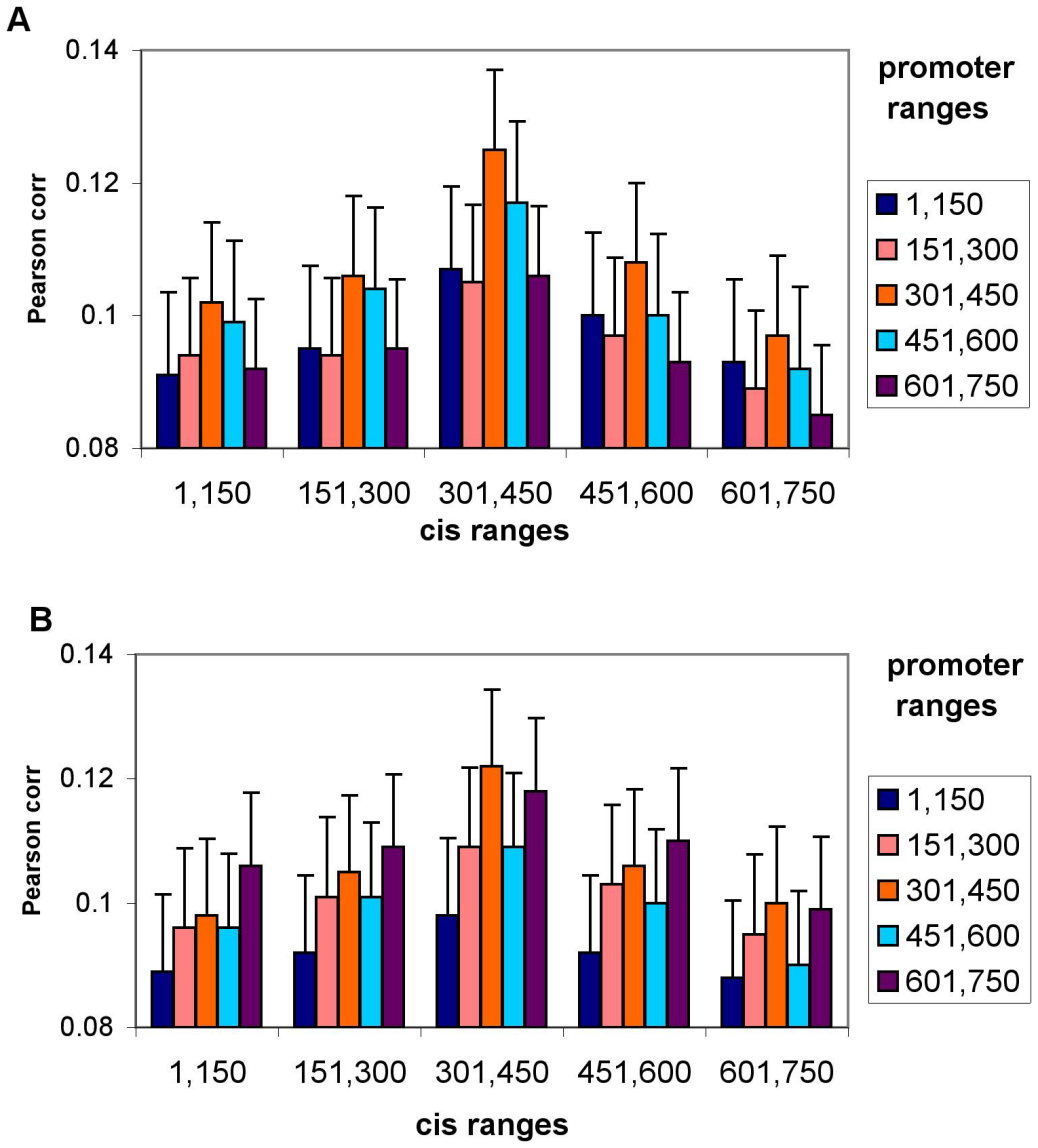
Range-wise Pearson correlations of the G2 patterns in the cis-promoter pairs as defined in Ref.^13^. The 750 nucleotide-long promoter and cis regions were divided into 5 equal ranges and only those G2s were counted where the regulatory region in question had G2s in the appropriate range. The correlation between the number of G2s in the promoter-cis pairs were calculated for each range pair separately. The error bars denote standard deviations. Correlation values for (A) minus strand-coded gene promoter-cis pairs; (B) plus strand-coded gene promoter-cis pairs.

**Figure 6:**
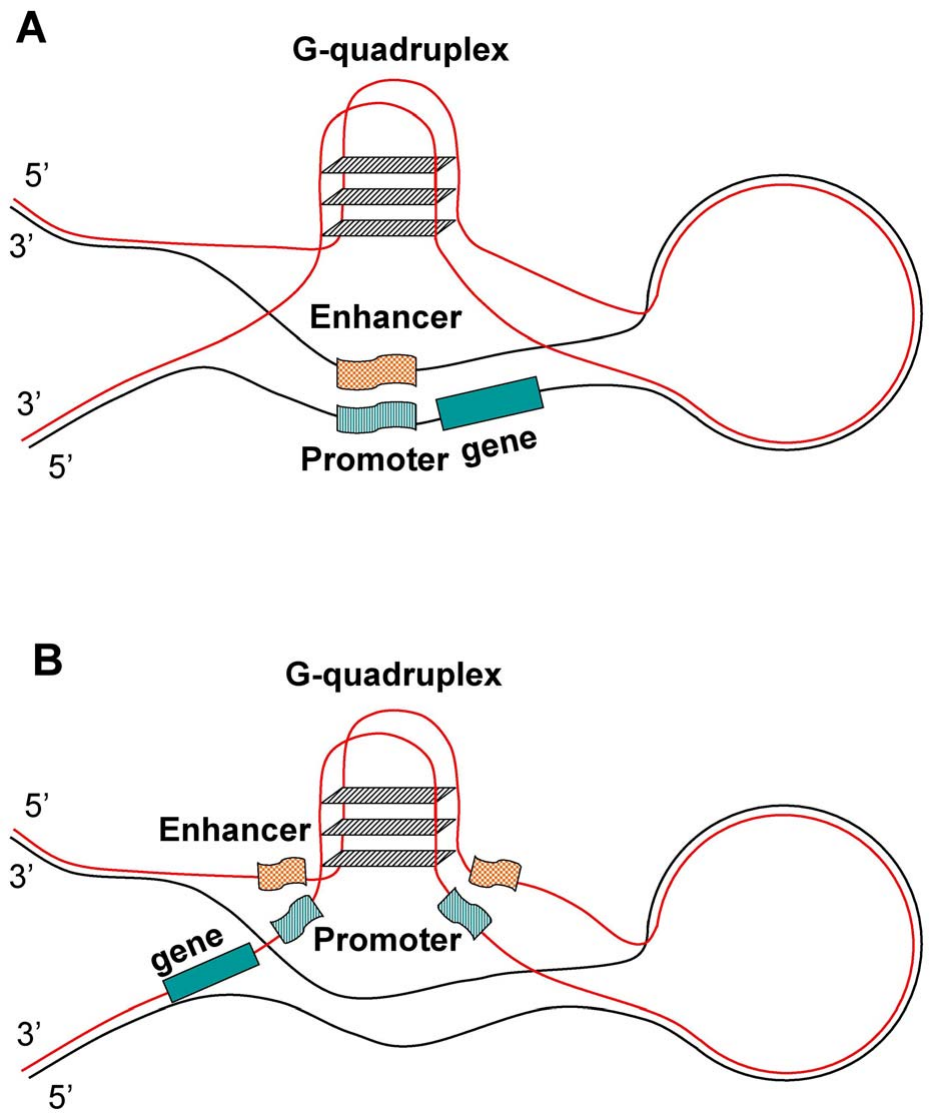
Models of enhancer-promoter binding facilitated by transiently forming G-quadruplexes. The gene is coded (A) on the (−) strand, (B) on the (+) strand. The large loop shows looping-out of double-stranded DNA representing the intervening region between the promoter and the enhancer.

To further explore the potential regulatory role of G2s we calculated the correlation of their numbers for various subsets of the correlated DHS promoter-cis pairs identified in Ref. 13. After dividing the 750-nt regions into five equal, 150 nt-long ranges we calculated the pairwise correlations of the number of G2s between the promoters and cis regions considering only those pairs that fall into the appropriate ranges. We calculated the correlation values separately for the negative and positive strand-coded genes (Figure 5). For the negative-strand coded genes the highest correlation is between the middle, i.e. the DHS ranges, indicating a potential interaction between the promoter and cis region G2s, whereas for the marginal ranges the correlations are significantly smaller, the smallest correlation shown between the most marginal ranges (i.e. between the 1–150 and the 601–750 ranges). This middle-range correlation (between the ranges of 301–450 in both the promoters and cis regions) is the highest of all pairwise range values. However, for the plus strand-coded genes the promoter 601–750 ranges (purple bars in Figure 6b) correlate the most (except for the middle cis range) with the cis ranges. Using Student's t-test revealed that all values are significantly different although for the plus-strand coded promoters (in Figure 5b) the middle-range promoter-cis values (the 301–450 range promoters paired with the 301–450 range cis elements) have only a marginally significant (p-value = 0.023) difference when compared to the 601–750 range promoters paired with the 301–450 range cis elements (i.e. middle orange bar vs. the middle purple bar in Figure 5b).

### G2s promote the binding of the cis regions (enhancers) and promoters

Figure 6 offers an explanation for the different tendencies in the cis region-promoter correlations in Figure 5. According to the model, for the negative strand-coded genes the G2s (Figure 6a) on the promoter and the cis region come into physical proximity on the positive strand and form a G-quadruplex together, which release the enhancer and promoter regions from their double-stranded Watson-Crick type base-pairing, making interaction possible between the two regulatory regions. However, for the positive strand-coded genes (Figure 6b) this kind of G-quadruplex forming would be less productive, as the G-quadruplex formation could prevent the binding of transcription factors making the DHS sites less, rather than more accessible for protein binding.

## Discussion

While promoters have been shown both *in silico* and by experiments to contain G-quadruplexes with specific roles in the regulation of gene expression, no such studies exist exploring the potential function of G-quadruplexes in distal regulatory elements. We analyzed their occurrence in these cis elements, relying on the set of 1.5 million DNase hypersensitive (DHS) cis-promoter pairs originally identified in Ref. 13. Both the cis elements and the promoters have a significantly higher occurrence of G4-forming motifs at the boundaries (especially at the 3′ boundaries) of the DHS regions, but relatively scarce in the actual DHS regions. This might be interpreted in two ways (not mutually exclusive): (i) the enrichment of G4-forming patterns at the DHS boundaries might have a specific role in chromatin remodeling, making the DHS regions more accessible for the transcription machinery as it was observed in Ref. 3; (ii) G4-forming patterns are scarce in the actual DHS regions because a G-quadruplex could prevent transcription as it was shown in Ref. 15.

We also found that the promoter regions show an asymmetric distribution of the G2s, depending on the strandedness of the genes and their promoters. While the promoters of the minus strand-coded genes showed a strong correlation with the cis regulatory elements (r = 0.709) over the extended DHS regions (defined for both the promoters and the cis elements as the exact 150 nt-long DHS regions extended by 300 nucleotides in both directions), the promoters of the plus strand-coded genes have a much smaller correlation value of 0.248 with the cis elements.

While the prevailing view is that G-quadruplexes have short loops containing maximum 7 nucleotides, it has been also found that a relatively stable G-quadruplex may still be formed with a central loop of 21 or even 30 nucleotides, assuming the two other loops are short^16^. Furthermore, the authors found that in these cases the melting temperature tends to become length-independent, showing that one cannot even propose an upper limit for loops size in vitro^16^. This is in accordance with our findings in general as every cis element – promoter pair was included in our analysis, up to a distance of 0.5 megabase (the upper limit in Ref. 13) i.e. we extended the middle loop to very long distances to cover in principle any distance between a promoter and a distal cis regulatory element.

Our model also supports the “looping” theory according to which promoter and enhancer come into physical proximity by the looping-out of the intervening DNA between them^17^. While experimental evidence supports this theory, it is not quite clear yet if the looping-out happens first and transcription factor binding is secondary or the other way around. As shown by Guo et al.^18^ for the immunoglobulin heavy-chain locus the looping and nuclear migration to the site of the transcription happen first, preceding transcription. While it is not clear yet if this model can be applied to promoter-enhancer binding and transcription activation in general, our model is in agreement with this limited experimental evidence.

As outlined in Figure 6, G-quadruplex forming between the enhancer (or cis regulatory element in general) and promoter DHS sites might play out differently for the template and non-template strands, making gene expression less productive for the template-strand forming G-quadruplexes. This might explain the different patterns of different cis-promoter ranges' G2 correlation in Figure 5. While for the minus strand-coded genes the promoter middle range (i.e. the actual DHS regions) correlates the most with the cis elements, for the plus strand-coded genes the highest correlation values in the promoters are shifted downstream, apparently making the actual promoter DHS regions less prone to G-quadruplex forming. Our model in Figure 6 would also explain the overall higher correlation values of G2s between the cis elements and the minus strand promoters shown in Figure 2.

Taken together, we have shown that the G-rich patterns observed at the boundaries of DNAse I hypersensitive promoters and cis regions can facilitate the formation of G-quadruplexes at these positions. We have shown that these patterns significantly correlate with transcription factor binding sites but seem to have further functionality. The range-wise correlations of G2s between promoters and their cis pairs is different for plus- and minus-stranded genes, which we explained with a model where a temporary G-quadruplex forming between the enhancer and promoter can facilitate their efficient binding. Our model is in accordance with the experimental evidence of the looping-out theory of enhancer-promoter binding and hopefully further contribute to the clarification of this important momentum of gene expression regulation.

## Methods

The human genome variant hg19 was downloaded from the NCBI website. The cis regulatory element-promoter pairs were taken from Ref. 13. Transcription factor binding sites for the entire human genome were taken from Ref. 14. To calculate the variance of the Pearson correlations between G2s in the different ranges of promoters and cis elements in Figure 5, we randomly selected 10,000 pairs in the ranges in question, recalculated the correlation for them and repeated the procedure 100 times. To make sure that the correlations were not the result of spurious coincidences we also repeated the procedure in a way that the promoter values were randomly paired with the cis elements, with the only restriction that they were taken from the appropriate ranges. In this latter randomization we got similar variance but the correlation values dropped to close to zero (r < 0.01). Data manipulation, including G- and C-rich pattern searches and statistical analyses were carried out by in-house Perl scripts that are available at request from the author.

## Supplementary Material

Supplementary Information Supplementary Figures and Table
