# Supplementary material for: Enhancer-promoter interaction facilitated by transiently forming G-quadruplexes
Source: Sci Rep. 2015 Mar 16;5:9165. doi: 10.1038/srep09165 (PMC4360481; doi:10.1038/srep09165)
Supplement: Supplementary Information — Supplementary Figures and Table [file srep09165-s1.doc]

# Enhancer-promoter interaction facilitated by transiently forming G-quadruplexes

Hedi Hegyi ^1^

^1^CEITEC—Central European Institute of Technology, Masaryk University, CZ-62500 Brno, Czech Republic. tel: +420 549 494 278

Correspondence to:  [hegyi@ceitec.muni.cz](mailto: hegyi@ceitec.muni.cz %0D%0C)

[**Supplementary Figures and Table**](mailto: hegyi@ceitec.muni.cz %0D%0C)

**Supplementary Figure 1**. Pearson correlations of G2 vs G4 pattern occurrences across the 750 positions in and around the DHS regions in (**A**) promoters; (**B**) cis regulatory elements.

**Supplementary Figure 2 (next page)**. Transcription factor binding sites (TFBSs) and G2 patterns in and around the cis regulatory elements. (**A**) Total number of TFBSs. (**B**) Total number of G2 patterns. (**C**) Scatterplot of values in (**A**) against values in (**B**), with the Pearson correlation value. (**D**) Position-wise ratios of all TFBSs and all G2s in the cis elements. (**E**) Position-wise reverse ratios, of G2s and TFBSs.

**Supplementary Table 1.** Pearson correlation values between transcription factor and G2 occurrences in various ranges of **(A)** cis regulatory elements and **(B)** promoters. The full regions contain the entire 750 nucleotides extended regions around the DHS regions of cis elements and promoters, mid 250 and mid 150 regions cover the middle regions of 250 and 150 nucleotides, respectively, the 5’ and 3’ regions cover the 5’ and 3’ boundaries of the DHS regions, respectively, and their immediate vicinity in a 100 nucleotide range.
